# RECQ1 Helicase Silencing Decreases the Tumour Growth Rate of U87 Glioblastoma Cell Xenografts in Zebrafish Embryos

**DOI:** 10.3390/genes8090222

**Published:** 2017-09-06

**Authors:** Miloš Vittori, Barbara Breznik, Katja Hrovat, Saša Kenig, Tamara T. Lah

**Affiliations:** 1 Department of Genetic Toxicology and Cancer Biology, National Institute of Biology, Večna pot 111, 1000 Ljubljana, Slovenia; barbara.breznik@nib.si (B.B.); tamara.lah@nib.si (T.T.L.); 2 Department of Biology, Biotechnical Faculty, University of Ljubljana, Večna pot 111, 1000 Ljubljana, Slovenia; hrovat.katja4@gmail.com; 3 International Postgraduate School Jozef Stefan, Jamova 39, 1000 Ljubljana, Slovenia; 4 Structural Biology Laboratory, Elettra-Sincrotrone Trieste, Strada Statale 14-km 163, 5, Basovizza, 34149 Trieste, Italy; sasa.kenig@fvz.upr.si; 5 Department of Biochemistry, Faculty of Chemistry and Chemical Engineering, University of Ljubljana, Večna pot 113, 1000 Ljubljana, Slovenia

**Keywords:** cancer, cell cycle, DNA damage, intravital imaging, RNA interference, theranostics

## Abstract

RECQ1 helicase has multiple roles in DNA replication, including restoration of the replication fork and DNA repair, and plays an important role in tumour progression. Its expression is highly elevated in glioblastoma as compared to healthy brain tissue. We studied the effects of small hairpin RNA (shRNA)-induced silencing of RECQ1 helicase on the increase in cell number and the invasion of U87 glioblastoma cells. RECQ1 silencing reduced the rate of increase in the number of U87 cells by 30%. This corresponded with a 40% reduction of the percentage of cells in the G2 phase of the cell cycle, and an accumulation of cells in the G1 phase. These effects were confirmed in vivo, in the brain of zebrafish (*Danio rerio*) embryos, by implanting DsRed-labelled RECQ1 helicase-silenced and control U87 cells. The growth of resulting tumours was quantified by monitoring the increase in xenograft fluorescence intensity during a three-day period with fluorescence microscopy. The reduced rate of tumour growth, by approximately 30% in RECQ1 helicase-silenced cells, was in line with in vitro measurements of the increase in cell number upon RECQ1 helicase silencing. However, RECQ1 helicase silencing did not affect invasive behaviour of U87 cells in the zebrafish brain. This is the first in vivo confirmation that RECQ1 helicase is a promising molecular target in the treatment of glioblastoma.

## 1. Introduction

RECQ1 helicase is the first member of the RecQ family to be discovered in humans, and may have multiple roles during DNA replication, including restoration of the replication fork and DNA repair [1,2,3]. It may also represent a good potential molecular target for the treatment of various types of cancer. For example, the silencing of RECQ1 helicase, using small interfering RNA (siRNA) delivered with liposomes into mouse hepatocellular carcinoma xenografts, proved effective in selectively inhibiting tumour growth, while having little effect on healthy liver tissue [4]. Furthermore, HeLa cells were shown to be more sensitive to irradiation when RECQ1 was experimentally silenced. It was found that RECQ1 silencing reduces the rate of DNA replication of HeLa cells, and results in cell cycle arrest [1]. Recently, it was also suggested that RECQ1 may have a role in the regulation of cell migration and invasion in two cervical and breast cancer cell lines [5], as reduced cell invasion and migration were observed in RECQ1-knockdown cells.

Glioblastoma is the most aggressive type of glioma, and also the most frequent among brain tumours [6,7]. In gliomas, the expression of RECQ1 is highly elevated, whereas it is low in healthy brain tissue, as demonstrated by immunohistochemical analysis of tissue samples from patients, making RECQ1 a potential molecular target for the treatment of this type of tumour [8]. Treatment of glioblastoma is, apart from surgery, based on DNA damage-inducing therapy with radiation and temozolomide [9]. Supplementing such therapy by selectively targeting enzymes involved in DNA repair in glioblastoma cells, such as RECQ1, is thus a perspective approach to improve treatment success. Mendoza-Maldonado et al. [8] showed that silencing RECQ1 expression with short interfering RNA and short hairpin RNA in the glioblastoma cell line T98G, increased the load of DNA damage in glioblastoma cells. Furthermore, RECQ1-silenced T98G and U87 glioblastoma cells were more susceptible to treatment with temozolomide [8].

Zebrafish are an emerging model in glioma research [10,11], and xenotransplantation of human glioblastoma has proven to be a successful approach. In previous studies, fluorescently labelled or fluorescent protein-expressing glioblastoma cells were implanted in zebrafish, in order to observe tumour-associated angiogenesis, to determine the effects of drugs, and to study the involvement of signalling pathways, such as Wnt signalling, in tumour progression [12,13,14,15]. One of the benefits of this model is that zebrafish embryos lack an adaptive immune system, obviating the need for immunosuppression [16]. Another advantage is the transparency of the embryos, which makes imaging of tumour progression at single cell resolution easier than in mammalian models [10,13,17]. Furthermore, the fecundity of zebrafish, and the low cost of maintenance and experimentation, are advantages of the zebrafish model, that holds great promise for its use in drug screening in vivo [18,19,20].

In the present study, we validated the effects of RECQ1 silencing on the growth of U87 cell xenografts in vivo in a zebrafish embryo model. When RECQ1-silenced U87 cells were implanted into the brain of zebrafish embryos, the effect of RECQ1 silencing on the tumour growth rate confirmed the biological effects demonstrated in experiments in vitro.

## 2. Materials and Methods

### 2.1. Ethical Statement

The experimental procedures were approved by the Republic of Slovenia National Medical Ethics Committee, approval No. 92/06/12. All procedures were performed according to the relevant regulations.

### 2.2. Preparation of RECQ1-Silenced Fluorescent Glioblastoma Cells

The U87-MG human glioblastoma cell lines were cultured in Dulbecco’s modified Eagle’s medium (Sigma-Aldrich, St. Louis, MO, USA) supplemented with 10% foetal bovine serum (Gibco, Grand Island, NE, USA), 0.02 mM l-glutamine (Sigma-Aldrich), 100 U/mL penicillin (Sigma-Aldrich), 0.1 mg/mL streptomycin (Sigma-Aldrich), 1 mM Na-pyruvate (Gibco), 0.1 mg/mL geneticin (Gibco), and non-essential amino acids (Sigma-Aldrich) at 37 °C and in an atmosphere with 5% CO_2_. Cells were transfected with the plasmid vector pCMV DsRed-Express2 to stably express the red fluorescent protein DsRed, as described previously [21].

For RECQ1 helicase silencing, the cells were transfected with the pLKO.1 lentiviral small hairpin RNA (shRNA) expression vector encoding a RECQ1 mRNA-targeting sequence (5′-GAGCTTATGTTACCAGTTA-3′), as described previously [2]. Control cells were transfected with the same vector encoding shRNA not homologous to any human sequence [22]. The success of RECQ1 silencing was validated by Western blot analysis, using α-tubulin as internal control. Western blot band intensity was quantified using *Analyze/Gels* in ImageJ [23].

### 2.3. Analysis of the Rate of Cell Number Increase In Vitro

RECQ1-silenced and control U87-MG cells were plated in 12 wells of 96-well plates, at a density of 3000 cells per well. Cells were imaged with a 4× objective, using green excitation at 3 h after seeding, followed by re-imaging at 1 day, 2 days, and 3 days after seeding, with an Eclipse TE300 inverted microscope (Nikon, Tokyo, Japan) with identical excitation intensity and camera settings.

For the quantification of cell number, we used *Analyze/Analyze particles* in ImageJ to obtain the integrated density values of individual cells. Based on these measurements, the average fluorescence intensity of individual cells at 3 h after seeding was calculated for each clone (i.e., control cells and RECQ1-silenced cells). We obtained the number of cells per imaging field by measuring the fluorescence intensity in images of cultured cells, and dividing the intensity by the average fluorescence intensity per cell of that clone. The relative increase in cell density was determined by dividing cell density at a given time point with cell density at 1 day after seeding. All experiments were performed in triplicates, with 12 wells per treatment.

Additionally, differences in the rate of increase in cell number were evaluated by direct counting. Cells were seeded (3 × 10^4^ cells/well) in a 24-well plate (Corning Costar, Corning, NY, USA). After incubation, they were trypsinized and stained with trypan blue (Sigma) to exclude dead cells. Cells were counted using a haemocytometer with an Eclipse TS100 inverted transmitted light microscope (Nikon). The counting was performed in triplicates of two wells per cell clone and per time of incubation (2 days and 3 days).

### 2.4. Cell Cycle Analysis

One million control cells and RECQ1-silenced cells were washed twice with cold phosphate buffered saline (PBS) and fixed in ice-cold 70% ethanol overnight at 4 °C. Then, cells were washed with PBS, stained with propidium iodide (50 µg/mL; Miltenyi Biotec, Bergisch Gladbach, Germany) with RNAse A (100 µg/mL; Sigma) and analysed using MACSQuant Analyzer 10 and MACSQuantify software (both from Miltenyi Biotec). The percentage of cells in G1, S, and G2 cell cycle phases were determined using FlowJo software (Tree Star Inc., Ashland, OR, USA) and the Dean–Jett–Fox modelling algorithm [24,25].

### 2.5. Analysis of Tumour Growth In Vivo

Wild type AB zebrafish (*Danio rerio*) were maintained at 28 °C in accordance with the Organization for Economic Cooperation and Development guidelines [26]. The zebrafish embryos were collected and incubated in reconstituted water [27]. To inhibit pigment formation, 0.005% (*w*/*v*) phenylthiourea was added to reconstituted water after 36 h of embryonic development.

For injection of DsRed-expressing U87 cells, culture medium was thoroughly removed with three changes of PBS, and cells were re-suspended in PBS at a concentration of 3 × 10^7^ cells per mL. Embryos, at 52 h after fertilization, were injected with 50–100 glioblastoma cells with the MICROINJECTOR system (Tritech Research, Los Angeles, CA, USA) using a borosilicate glass capillary. After cell implantation, embryos were incubated in reconstituted water at 31 °C in 48-well plates. Xenografts were imaged with an Eclipse TE300 inverted microscope (Nikon) at 1 day, 2 days, and 3 days after implantation in embryos lying on their sides. The fluorescence intensity of xenografts was quantified in ImageJ by selecting the region of interest corresponding to the glioblastoma tumour on the basis of a fixed pixel intensity threshold value, and measuring the integrated density of the region of interest. The relative increase in fluorescence intensity, as a measure of tumour growth, was determined by dividing the measured values by the fluorescence intensity of the tumour in the same embryo at 1 day after implantation. All experiments were performed in triplicate, with 20 embryos per treatment.

### 2.6. Analysis of Cell Invasion In Vivo

Invasion of U87 cells from glioblastoma xenografts was measured in images of embryos in lateral orientation, as described previously [28]. Briefly, tumour length (largest dimension) and width (perpendicular to the largest dimension) were measured and averaged. Measurements obtained at day 3 after implantation were divided by measurements at day 1 after implantation, to obtain a relative glioblastoma cell invasion from the tumour between these two timepoints.

### 2.7. Statistical Analyses

Pairwise comparisons between RECQ1-silenced and control cells were performed with the Mann–Whitney U test. Tumour growth between days 2 and 3 after implantation was tested with the Friedman test. Statistical analyses were performed in GraphPad Prism version 5.01 (GraphPad Software, La Jolla, CA, USA).

## 3. Results

### 3.1. Reduced Rate of U87 Cell Number Increase as a Result of RECQ1 Helicase Silencing In Vitro

**Figure 1:**
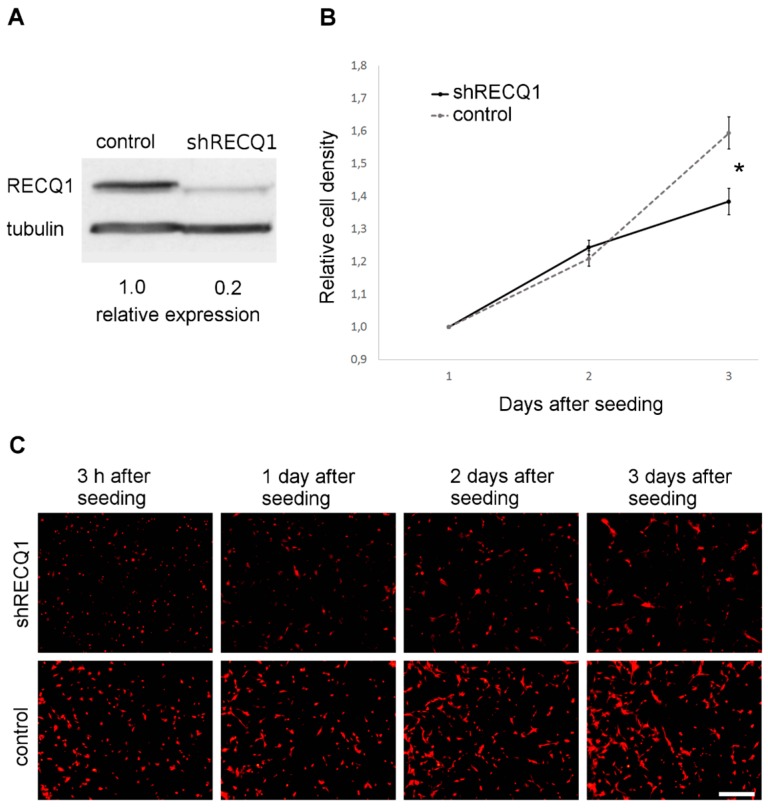
RECQ1 silencing in U87 DsRed cells and its effects on the increase in cell number in vitro. (**A**) The expression of RECQ1 helicase in U87 cells transfected with small hairpin RNA (shRNA) targeting RECQ1 (shRECQ1) and control cells as quantified by Western blot analysis. The relative expression of RECQ1 in RECQ1-silenced cells was 20% of its expression in the control cells; (**B**) Changes in cell density of cultured RECQ1-silenced (shRECQ1) and control U87 DsRed cells as determined by measurement of DsRed fluorescence intensity. The increase in the number of RECQ1-silenced cells was reduced relative to the control cells on day 3 after seeding. Means ± standard error (SE) are shown; (**C**) Sequential images of U87 DsRed cells in adherent cultures, captured at 3 h after seeding and at the same time during the following three days. Scale bar: 400 µm. Significance: * = *p* ≤ 0.05.

**Figure 2:**
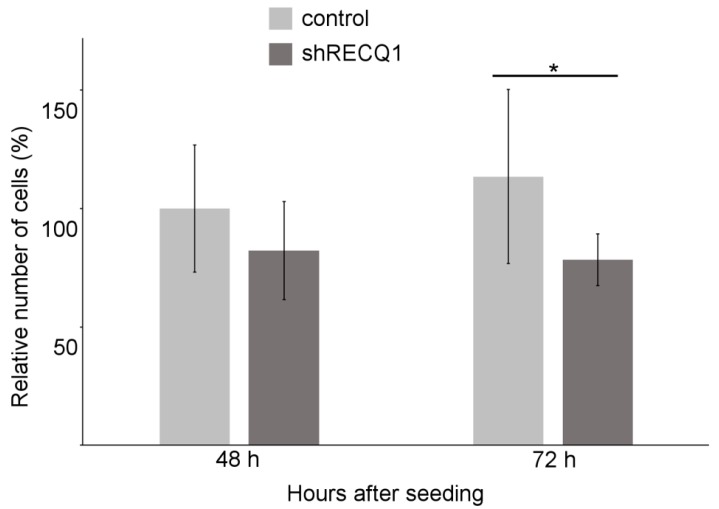
Differences in the rate of increase in cell number between RECQ1-silenced and control U87 DsRed cells as determined by direct counting at 2 days and 3 days after seeding. Significant differences (* = *p* ≤ 0.05) in cell number were found at 72 h after seeding.

Transfection with shRNA targeting RECQ1 resulted in an 80% decrease in RECQ1 expression at the protein level (Figure 1A). In vitro, U87 cells rapidly increased in number during the period between day 1 after plating and day 3 after plating (Figure 1B,C). Measurements of relative changes in cell density in 96-well plates, estimated from changes in measured DsRed fluorescence intensity, demonstrated that the increase in the number of RECQ1-silenced U87 cells was reduced by approximately 30%, as compared to the control cells (Figure 1B). The difference was significant at day 3 after seeding, with control cells increasing in density by 59 ± 5% and RECQ1-silenced cells by 38 ± 4% relative to day 1 after seeding. A smaller increase in cell number between RECQ1-silenced cells and control cells was confirmed with direct counting, using a haemocytometer (Figure 2).

### 3.2. Cell-Cycle Perturbation in U87 Cells Resulting from RECQ1 Helicase Silencing

**Figure 3:**
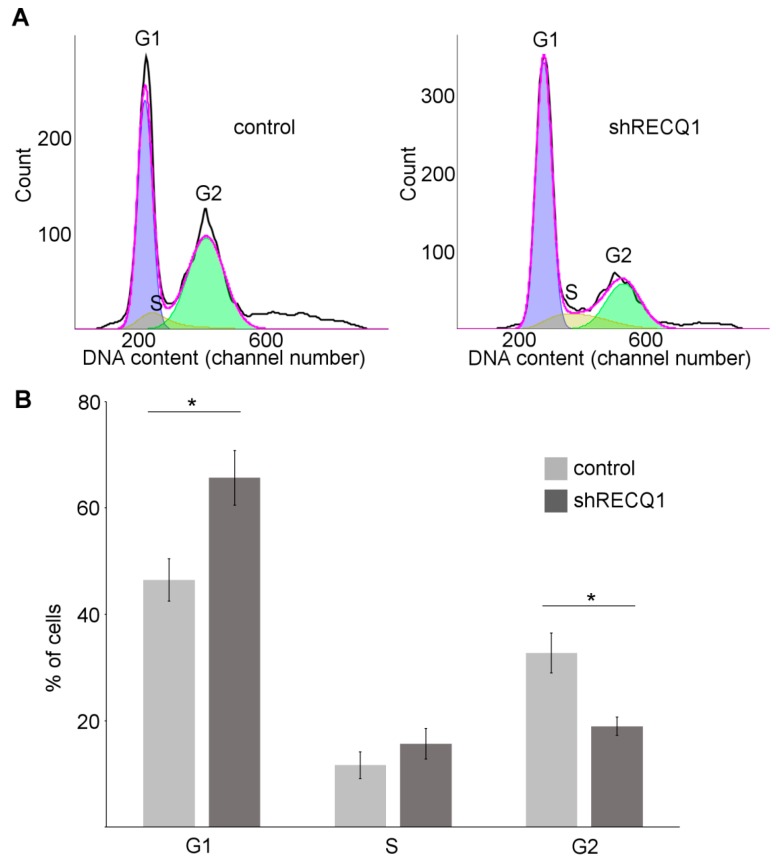
RECQ1 silencing resulted in cell cycle perturbation in U87 DsRed cells. (**A**) Typical histograms of cells in the G1, S, and G2 phases of the cell cycle as obtained with flow cytometry after propidium iodide staining. The population of cells in the G2 phase is less numerous in RECQ1-silenced cells; (**B**) Summary of cell cycle analyses. RECQ1-silenced U87 cells showed a larger percentage of cells in the G1 phase and a smaller percentage of cells in the G2 phase, whereas there was no significant difference in the percentage of cells in the S phase. Means ± SE are shown. Significance: * = *p* ≤ 0.05.

Cell cycle analysis using flow cytometry demonstrated that RECQ1-silenced U87 cells show a larger percentage of cells in the G1 phase (65 ± 5%) of the cell cycle, and a corresponding smaller percentage of cells in the G2 phase (19 ± 2%), as compared to control cells (46 ± 4% cells in G1 phase and 33 ± 4% cells in G2 phase). No statistically significant differences in the percentage of cells in the S phase were observed between RECQ1-silenced and control cells (Figure 3). 

### 3.3. Absence of Tumour Growth in U87 Cell Xenotransplantsin the Zebrafish Yolk Sac

**Figure 4:**
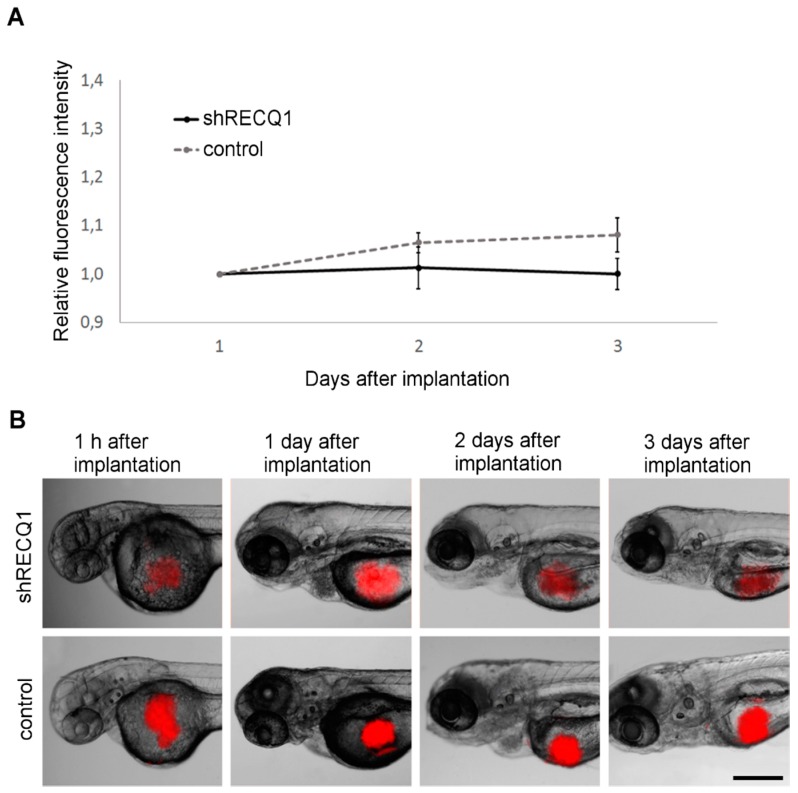
Xenotransplantation of U87 DsRed cells in the yolk sac of zebrafish embryos. (**A**) Changes in DsRed fluorescence in the yolk sac between day 1 and day 3 after implantation. Means ± SE are shown. No significant increase in fluorescence intensity was observed in RECQ1-silenced or in the control U87 cells; (**B**) Sequential images of xenotransplants in the yolk sac of zebrafish embryos between 1 h and 3 days after implantation. Scale bar: 250 µm.

In the first experiment, 50–100 cells were injected in the yolk sac of each zebrafish embryo. The fluorescence emitted by the xenografts was measured at day 1 and day 3 after implantation, in order to calculate the relative increase in fluorescence signal. When implanted into the yolk sac, there was no increase in fluorescence intensity, neither in RECQ1-silenced cells nor in control cells between day 1 and day 3 after implantation (Figure 4), although RECQ1-silenced cells and control cells were able to survive in the yolk sac.

### 3.4. In Vivo Observation of Xenotransplant Growth in the Zebrafish Embryonic Brain

**Figure 5:**
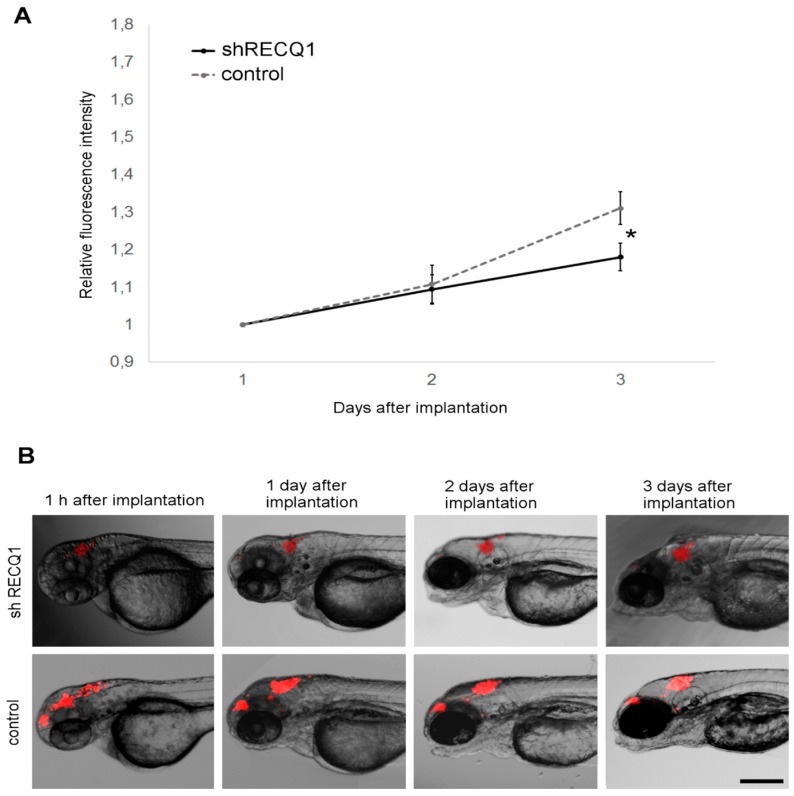
Xenotransplanted U87 DsRed cells in the brain of zebrafish embryos. (**A**) Changes in DsRed fluorescence of xenografts in the brain of zebrafish embryos between day 1 and day 3 after implantation. Averages ± SE are shown. The increase in fluorescence intensity of RECQ1-silenced cells was significantly smaller relative to control cells at day 3 after seeding; (**B**) Sequential images of xenotransplants in the brain of zebrafish embryos between 1 h and 3 days after implantation. Scale bar: 250 µm. Significance: * = *p* ≤ 0.05.

When U87 cells were implanted in the brain of zebrafish embryos, the resulting tumours grew rapidly between day 1 and day 3 after implantation, indicating that the brain is a more suitable environment to study tumour growth (Figure 5). Relative changes in DsRed fluorescence intensity in RECQ1-silenced U87 cells were approximately 30% lower than in the control cells on day 3 after implantation (Figure 5A). Fluorescence intensity relative to day 1 after implantation increased by 18 ± 4% in RECQ1-silenced xenografts, and 31 ± 4% in control xenografts, at 3 days after implantation.

### 3.5. Effects of RECQ1 Silencing on Invasion of U87 Cells in the Embryonic Brain

**Figure 6:**
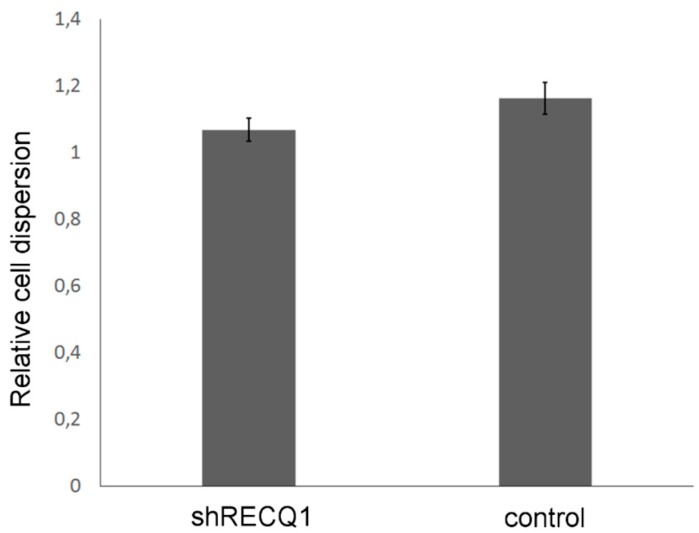
Relative invasion of RECQ1-silenced and control U87 cells between day 1 and day 3 after implantation. No significant differences in the rate of invasion were found. Means ± SE are shown.

To assess whether RECQ1-silencing affects invasion of U87 cells, we determined the rate of invasion from the tumour between day 1 and day 3 after implantation by measuring distances between marginal cells of the tumours as a measure of relative cell dispersion. Cells invaded the brain of zebrafish embryos, resulting in 10–20% increase in distances between marginal tumour cells during observation (Figure 6). However, the rates of invasion did not differ significantly between RECQ1-silenced U87 xenografts and control xenografts, indicating that RECQ1 silencing did not affect invasion of U87 cells.

## 4. Discussion

In this study, we demonstrated that RECQ1 silencing reduces the rate increase in the number of U87 cells in vitro, which is likely the result of cell cycle perturbation by RECQ1 silencing and reduced proliferation. Alternatively, an increase in the frequency of cell death may lead to a slower increase in cell number, which cannot be excluded entirely.

Our results are in agreement with a previous study on the glioblastoma cell lines U87, T98G, and IMR-90, in which RECQ1 was silenced with the use of siRNA. Upon RECQ1 silencing, these cell lines displayed a decrease in their colony-forming ability, which was linked to cell cycle perturbation and a decrease in BrdU incorporation in the cell line T98G, strongly suggesting that RECQ1 silencing reduces the rate of glioblastoma cell proliferation [8]. RECQ1 helicase silencing also inhibited DNA synthesis and cell cycle progression of the cervical cancer cell line HeLa, and the breast cancer cell line MDA-MB-231 [1]. We found that RECQ1 silencing affects the cell cycle of U87 cells, with more cells arrested in G1 phase and less cells in G2 phase as compared to control cells. Cell cycle arrest in G1 may be caused by accumulating DNA damage resulting from the inability of RECQ1-silenced cells to efficiently repair DNA damage. A greater load of DNA damage was previously demonstrated in RECQ1-silenced T98G glioblastoma cells as compared to control cells [8]. Unlike the hepatocellular carcinoma cell lines SK-HEP-1, KYN-2 and KYN-3, in which RECQ1 depletion was suggested to result in mitotic catastrophe due to accumulated DNA damage while cells divide [4], U87 cells apparently do not proceed to G2, and do not undergo cell division at all. This may be due to the preservation of the G1/S checkpoint in U87 cells, which are p53 wild type [28]. Different effects of RECQ1 depletion were previously observed in various cell lines. RECQ1 silencing in HeLa cervical cancer cells brought about a shift towards cell fractions in G2 and M phases [1]. By contrast, a shift towards the cell fraction arrested in the G1 phase was observed in T98G glioblastoma cells [8], as well as in the breast cancer cells MDA-MB-231 [5]. A transcriptome analysis showed that RECQ1 knockdown in cells leads to the downregulation of several genes involved in cell cycle progression, such as *CDK6*, *DICER1*, and *YES1*, in HeLa cells and MDA-MB-231 cells [5], which may explain the observed effects of RECQ1 silencing on cell cycle progression.

We then examined the effect of RECQ1 silencing in U87 on tumour growth in vitro, by implanting RECQ1-silenced and control U87 cells in the brain of zebrafish embryos, and found a corresponding decrease in the rate of tumour growth of RECQ1-silenced U87 xenografts. This is the first in vivo confirmation of the effects of RECQ1 silencing on glioblastoma cells. The silencing of RECQ1 is a promising potential treatment approach in gliomas, as its expression is elevated in this type of tumour, but is low in healthy brain tissue [8,29]. In addition, RECQ1 has an important role in cellular response to DNA damage, which results from current approaches to glioblastoma treatment targeting proliferating cells, including radiation and temozolomide. Indeed, RECQ1-silenced T98G and U87 cells were shown to be more susceptible to the cytotoxic effects of temozolomide, an alkalizing agent that causes DNA damage. This effect is likely linked to the functions of RECQ1 in DNA repair in malignant cells [8]. Additionally, RECQ1-targeting may assist potential novel treatment approaches, such as topoisomerase inhibition. Previous studies have shown that RECQ1 silencing results in greater sensitivity of the osteosarcoma U2-OS cell line to topoisomerase inhibitors such as camptothecin and etoposide. It was indicated that RECQ1 plays an important role in the restoration of replication forks upon their reversal, which results from topoisomerase inhibition by chemotherapeutics such as etoposide [2]. The inhibition or silencing of RECQ1 can thus contribute to the cytotoxic effects of topoisomerase inhibitors. This effect is particularly relevant to the targeting of glioblastoma stem-like cells. A transcriptomic study of neurospheres enriched in glioblastoma stem-like cells and the glioblastoma stem-like cell line NCH421k [30] showed that topoisomerase IIβ was highly upregulated in this cell line. While NCH421k cells were also found to be much more resistant to chemotherapeutics, such as cisplatin and methyl-methanesulfonate, as compared to several established glioblastoma cell lines, the silencing of topoisomerase IIβ expression increased the sensitivity of NCH421k cells to these agents. This suggests that this enzyme plays an important role in therapy resistance of glioblastoma stem-like cells [30].

Taken together, our results confirm that the effects of RECQ1 silencing observed in vitro correlate well with its effects in vivo, reducing tumour growth and perturbing the cell cycle. However, RECQ1 silencing had no effect on the invasion of U87 cells in vivo. This indicates that RECQ1 silencing by RNA interference is indeed a promising approach for glioblastoma treatment that may be combined with chemo- and radiation therapy, and could possibly increase their efficacy. Our results encourage future in vivo validations of novel therapeutic approaches in combination with RECQ1 silencing in glioblastoma using the zebrafish embryo model, as well as the use of zebrafish in validation of novel therapies for brain tumour treatment.
